# Supplementary material for: Virus Prevalence in Egg Samples Collected from Naturally Selected and Traditionally Managed Honey Bee Colonies across Europe
Source: Viruses. 2022 Nov 3;14(11):2442. doi: 10.3390/v14112442 (PMC9692946; doi:10.3390/v14112442)
Supplement: Supplementary file 1 [file viruses-14-02442-s001.zip › viruses-1995260-supplementary.pdf]

Supplementary information

Supplementary Table S1: Primer sequences

Overview of the primers used in this study.

Virus	Primers	Sequence '5-'3	Amplicon size (bp)	Ref
ABPV complex	ABPV-F	5'-TCA TAC CTG CCG ATC AAG-3'	197	[126]
	ABPV-R	5'-CTG AAT AAT ACT GTG CGT ATC-3'		
SBV	SBV-F	5'-TTG GAA CTA CGC ATT CTC TG-3'	335	[127]
	SBV-R	5'-GCT CTA ACC TCG CAT CAA C-3'		
BQCV	BQCV-F	5'-AGT GGC GGA GAT GTA TGC-3'	294	[127]
	BQCV-R	5'-GGA GGT GAA GTG GCT ATA TC-3'		
DWV-A	DWV-F	5'-TTC ATT AAA GCC ACC TGG AAC ATC-3'	136	[128]
	DWV-R	5'-TTT CCT CAT TAA CTG TGT CGT TGA-3'		
DWV-B	VDV-F2	5'-TAT CTT CAT TAA AAC CGC CAG GCT-3'	140	[129]
	VDV-R2a	5'-CTT CCT CAT TAA CTG AGT TGT TGT C-3'		
DWV complex	DWV_F8688	5'-GGT AAG CGA TGG TTG TTT G-3'	143	[130]
	DWV_B8794 R	5'-CCG TGA ATA TAG TGT GAG G-3'		
β-actin	ACTINE-F	5'-CGT GCC GAT AGT ATT CTT G-3'	271	[71]
	ACTINE-R	5'-CTT GTC ACC AAC ATA GG-3'		

Supplementary Figure S1: Comparison PCR and qRT-PCR

Comparison between gel based RT-PCR and qRT-PCR. All selected samples were positive on RT-qPCR for the represented virus in a 10^1-10^8 range / 10 eggs. For each virus, a positive and negative control is included. Each PCR included cDNA from 200 ng of RNA as described in the materials and methods section.

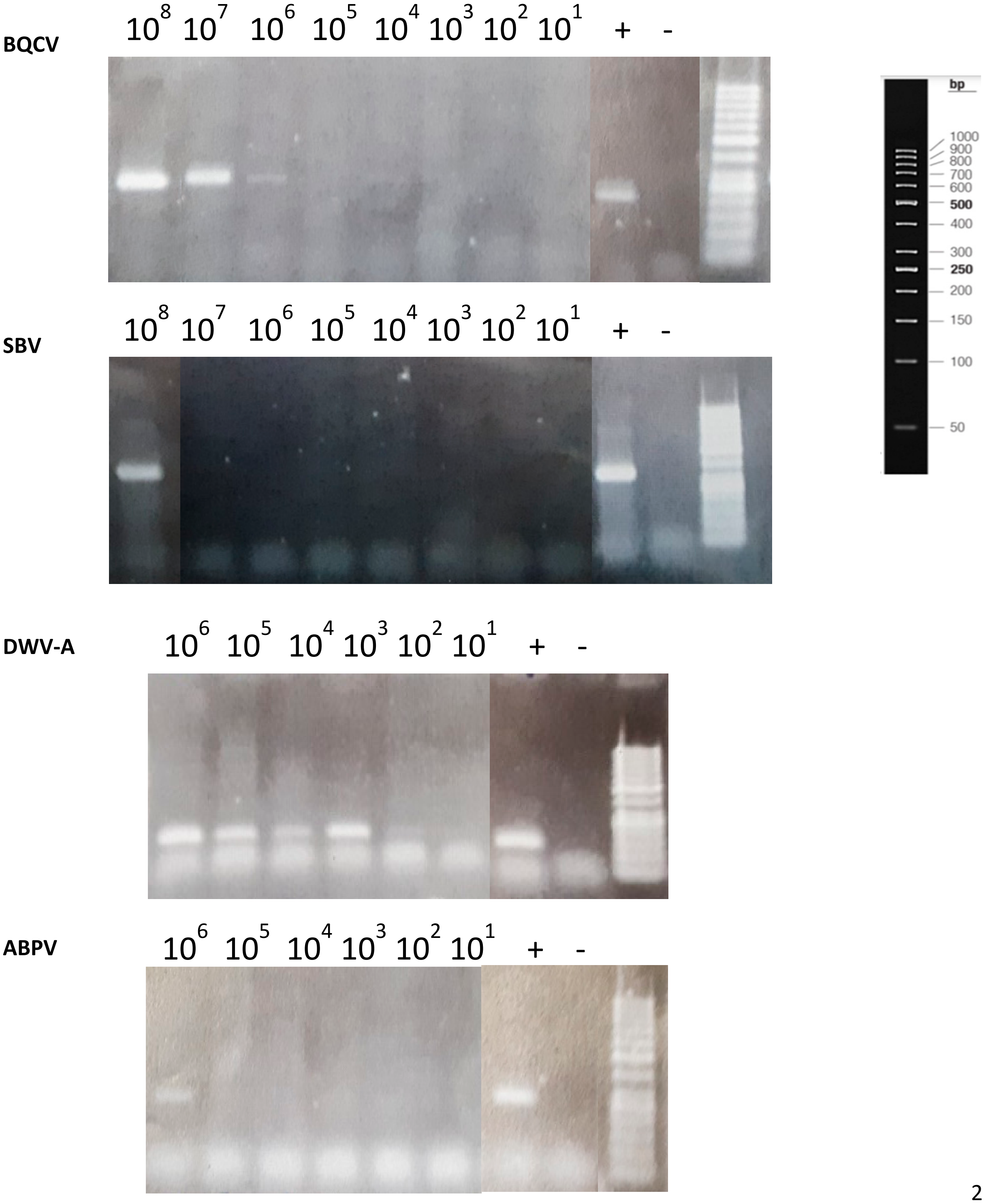

Supplementary Table S2: Additional information European Screening

Overview of supplementary information on the samples collected from each country.

Country	Sampling season	Subspecies	Nr of worker eggs on total number of samples	Colony health remarks	Location of sampled populations	Year of establishment of NSC
Belgium	Spring	*A.m. mellifera*	0 of 21	Signs of SBV	NSC: Bosland<br>TMC: Tessenderlo	2019
Croatia	Summer	*A.m. carnica*	0 of 10		TMC: Gorica	
France	Spring	*A.m. carnica, buckfast and* ligustica	0 of 26	1 colony with European foulbrood	NSC: Gragnague, Verfeil, Garidech, Puchin<br>TMC: Orgueil, Beaudésert	2018
Netherlands	Spring and summer	*A.m. mellifera*	0 of 16		NSC: Lelystad<br>TMC: Lelystad	2008
Norway	Summer	*Hybrids between A.m. buckfast and carnica*	16 of 20		NSC: Ask, Reinertsen<br>TMC: Skutelandet, Borgerskogen	1997
Portugal	Spring	*A.m. iberiensis*	0 of 10		TMC: Girmonde	
Romania	Spring	*A.m. carpatica*	0 of 13		NSC: Palocsay Cluj Napoca<br>TMC: Palacay, USAMV, Salaj	2019
Slovenia	Spring	*A.m. carnica*	0 of 72		TMC: across Slovenia	
Spain	Spring	*A.m. iberiensis*	0 of 10		TMC: Arbeiza	
Sweden	Summer	hybrids between *A.m. carnica, ligustica* and *mellifera*	18 of 18	signs of DWV, chalkbrood and possibly EFB	NSC: Näsudden (also referred to as Gotland bees)<br>TMC: Sigarve	1999
